# Gene expression profile analysis of human hepatocellular carcinoma using SAGE and LongSAGE

**DOI:** 10.1186/1755-8794-2-5

**Published:** 2009-01-26

**Authors:** Hui Dong, Xijin Ge, Yan Shen, Linlei Chen, Yalin Kong, Hongyi Zhang, Xiaobo Man, Liang Tang, Hong Yuan, Hongyang Wang, Guoping Zhao, Weirong Jin

**Affiliations:** 1 Department of Microbiology and Microbial Engineering, School of Life Sciences, Fudan University, Shanghai 200433, PR China; 2 International Cooperation Laboratory on Signal Transduction, Eastern Hepatobiliary Surgery Institute, Second Military Medical University, Shanghai 200438, PR China; 3 Department of Mathematics and Statistics, South Dakota State University, Brookings, SD 57006, USA; 4 National Engineering Center for Biochip at Shanghai, Shanghai 201203, PR China; 5 Chinese National Human Genome Center at Shanghai, 351 Guo Shou-Jing Road, Shanghai 201203, PR China; 6 Center for Clinical Pharmacology, Third Xiangya Hospital, Central South University, Changsha 410013, PR China; 7 Department of Hepatobiliary Surgery, General Hospital of Air Force PLA, Beijing 100036, PR China

## Abstract

**Background:**

Hepatocellular carcinoma (HCC) is one of the most common cancers worldwide and the second cancer killer in China. The initiation and malignant transformation of cancer result from accumulation of genetic changes in the sequences or expression level of cancer-related genes. It is of particular importance to determine gene expression profiles of cancers on a global scale. SAGE and LongSAGE have been developed for this purpose.

**Methods:**

We performed SAGE in normal liver and HCC samples as well as the liver cancer cell line HepG2. Meanwhile, the same HCC sample was simultaneously analyzed using LongSAGE. Computational analysis was carried out to identify differentially expressed genes between normal liver and HCC which were further validated by real-time quantitative RT-PCR.

**Results:**

Approximately 50,000 tags were sequenced for each of the four libraries. Analysis of the technical replicates of HCC indicated that excluding the low abundance tags, the reproducibility of SAGE data is high (R = 0.97). Compared with the gene expression profile of normal liver, 224 genes related to biosynthesis, cell proliferation, signal transduction, cellular metabolism and transport were identified to be differentially expressed in HCC. Overexpression of some transcripts selected from SAGE data was validated by real-time quantitative RT-PCR. Interestingly, sarcoglycan-ε (SGCE) and paternally expressed gene (PEG10) which is a pair of close neighboring genes on chromosome 7q21, showed similar enhanced expression patterns in HCC, implicating that a common mechanism of deregulation may be shared by these two genes.

**Conclusion:**

Our study depicted the expression profile of HCC on a genome-wide scale without the restriction of annotation databases, and provided novel candidate genes that might be related to HCC.

## Background

Being a worldwide malignant liver tumor, hepatocellular carcinoma (HCC) ranks the fifth in frequency among common human solid tumors and the fourth leading cause of cancer-related death [1,2]. The majority of HCC cases occur in Asia and Sub-Saharan Africa, but incidence has been increasing in Western Europe and the United States in recent years [3,4]. In China, HCC is now the second cancer killer [5]. Numerous studies have been carried out in an effort to elucidate molecular mechanism of hepatocarcinogenesis, metastasis and/or prognosis. Multiple genes have been reported to be involved in the development of HCC. For instance, mutations of *p53*, *β-catenin *and *AXIN1 *[6-10], activation of oncogenes such as *c-myc*, *c-met*, *c-jun*, *N-ras *and *nuclear factor κB *[11-16], and up-regulation of a set of genes including *GPC3*, *LCN2*, and *IGFBP-1 *were observed in a certain number of HCC cases [17-19]. These studies contributed greatly to our understanding of HCC in terms of individual molecules.

It is well known that the initiation and malignant transformation of cancer result from accumulation of genetic changes in the sequences or expression level of cancer-related genes. Thus it is of particular importance to determine gene expression profiles of cancers on a global scale. Several techniques have been developed for this purpose. Serial Analysis of Gene Expression (SAGE) allows quantitative measurement of gene expression profile through sequencing of short tags. Compared with microarrays, the expression profiles obtained by SAGE is exploratory in nature as it is not restricted to available annotations. It has been widely used in cancer studies since it was first introduced by Velculescu *et al*. in 1995 [20]. Saha *et al*. further developed LongSAGE which detects 21-bp tags instead of the original 14-bp tags, thus making it feasible to directly map the LongSAGE tags to genomic sequence data [21].

In the present study, we employed SAGE to comprehensively analyze gene expression profiles of normal human liver, HCC and the liver cancer cell line HepG2. The same HCC sample was further analyzed simultaneously using LongSAGE. To the best of our knowledge, there is no SAGE or LongSAGE expression profile of HCC available in the public domain. Our study provided a wealth of information on HCC expression profile, and comparative analysis of datasets of HCC vs normal liver led to the identification of a series of differentially expressed genes. The following real-time quantitative RT-PCR analysis further identified novel candidate genes potentially involved in hepatocarcinogenesis of HCC.

## Methods

### Cell culture and tissue samples

Human HCC cell line HepG2 was cultured in Dulbecco's modified Eagle's medium (Invitrogen, Carlsbad, CA) with 10% fetal bovine serum under 5% CO_2 _in a humidified incubator at 37°C. Cells were harvested at 80–90% confluence. Normal liver tissue was obtained from a patient who underwent hepatectomy because of hepatic hemangioma. Clinical HCC samples and corresponding non-tumorous liver tissues were derived from HCC patients and kept frozen in liquid nitrogen immediately after separation. All samples were collected with informed consent in accordance with the standards of Institutional Human Subjects Protection Review Board.

### SAGE and LongSAGE

Total RNAs were extracted from HepG2 and liver tissues using TRIzol reagent (Invitrogen, Carlsbad, CA) and then treated with DNaseI (Rhoch Diagnostics, Almere, The Netherlands) according to the manufacture's protocol. Polyadenylated mRNAs were then isolated using Oligotex Direct mRNA Midi Kit (QIAGEN). Three SAGE libraries were constructed starting with 200 ng polyA^+ ^mRNA from HepG2, normal liver tissue and HCC tissue respectively according to SAGE protocol [20,22]. LongSAGE was performed with the same amount of polyA^+ ^mRNA from the same HCC tissue used in SAGE following the procedures described previously [21]. Sequencing of SAGE and LongSAGE tags were carried out using BigDye Terminator Cycle Sequencing Kits and ABI 3700 DNA Sequencers (Applied Biosystems, Foster City, CA). The sequence and frequency of 14-bp tags from SAGE and 21-bp tags from LongSAGE were extracted from the raw sequence files of concatenated di-tags using SAGE2000 analysis software version 4.5 (kindly provided by Dr. K. Kinzler, Johns Hopkins University School of Medicine).

### Computational Analysis

To exclude sequencing errors, only tags detected at least twice in the four SAGE libraries (three SAGE libraries and one LongSAGE library) were included for further analysis. SAGEmap reliable mapping (, UniGene Build #182) [23] was used to establish tag-to-gene assignments. Comparison analysis between SAGE libraries was performed using the IDEG6 software [24]. Differentially expressed tags were selected according to the cut-off threshold (*p *value < 0.05, fold change > 3) calculated according to pairwise comparison algorithm [25]. The Cluster and Treeview programs were used to generate the average linkage hierarchical clustering and visualize changes of gene expression [26]. Functional classification of genes was performed using DAVID program  obtained from NIAID (National Institute of Allergy and Infectious Disease).

### Real-time quantitative RT-PCR

Total RNAs were extracted from HCC samples and corresponding nontumorous liver tissues using TRIzol reagent (Invitrogen) followed by treating with DNaseI according to the manufacture's protocol. 2 μg of total RNA was reverse transcribed in a total volume of 20 μl containing 200 units of SuperScript II RNase H^- ^Reverse Transcriptase (Invitrogen), 50 mM Tris-HCl (pH8.3), 75 mM KCl, 3 mM MgCl_2_, 10 mM dithiothreitol, 500 μM dNTPs each, 500 ng oligo(dT)_23 _primer and 40 units of RNaseOUT at 42°C for 50 min, followed by inactivating at 70°C for 15 min.

For real-time quantitative RT-PCR, 1 μl of the first strand cDNA and 5 μl of 2× SYBR Green PCR mix (Applied Biosystems, Foster City, CA) was added to a final volume of 10 μl. The thermal cycles were performed on LightCycler (Roche) following conditions at 95°C for 30 s, 40 cycles at 95°C for 5 s, 60°C for 5 s and 72°C for 30 s. Each reaction was performed in triplicate. The threshold cycle (Ct) value was used to calculate the expression ratio of target genes to internal control gene (*GAPDH*) with the formula 2^Ct(GAPDH)-Ct(target gene)^. Two-side student's *t *test was applied in the statistical analysis of quantitative RT-PCR data.

All the sequences of differently expressed transcripts were obtained from GenBank . PCR Primers were designed using Primer 3.0 . One set of primers were also designed for *GAPDH *which is considered an internal control for RT-PCR. The sequences of primers for each transcript were listed in Table 1.

**Table 1:** Sequences of primers used in RT-PCR

**Unigene**	**GenBank** **Accession No.**	**Product** **Size**	**Primers**
GAPDH	NM_002046	106 bp	F:ATGGGTGTGAACCATGAGAAG R:AGTTGTCATGGATGACCTTGG
CCL20	BC020698	200 bp	F:GCGCAAATCCAAAACAGACT R:CAAGTCCAGTGAGGCACAAA
S100P	BC006819	116 bp	F:TACCAGGCTTCCTGCAGAGT R:AGCCACGAACACGATGAACT
PEG10	NM_015068	111 bp	F:TCCTGTCTTCGCAGAGGAGT R:TTCACTTCTGTGGGGATGGA
SGCE	NM_003919	220 bp	F:ATGCAAACACCAGACATCCA R:TCTGATGTGGCAAGTTCTGC
XAGE-1	AF251237	159 bp	F:TCTGCAAGAGCTGCATCAGT R:AGCTTGCGTTGTTTCAGCTT
COL4A1	NM_001845	228 bp	F:ACGGGGGAAAACATAAGACC R:TGGCGCACTTCTAAACTCCT
ZFYVE1	NM_178441	213 bp	F:AACTGCTACGAAGCCAGGAA R:GTTGTGGCAGTGGAGGATTT
ZNF83	BC050407	224 bp	F:TGGGAAGGTCTTCGGTCTAA R:CGGCATGAAATCTCTGATGA
TNPO2	NM_013433	213 bp	F:GGCGTTGTGCAGGACTTTAT R:GGCAGTCTCCATGATCACCT
TM4SF1	NM_014220	169 bp	F:AGGGCCAGTACCTTCTGGAT R:AAGCCACATATGCCTCCAAG

## Results

### Summary of SAGE data

A total of 56,984, 63,800 and 47,149 tags were detected from the normal liver, HCC and HepG2 SAGE libraries respectively, and 51,632 tags were obtained from the HCC LongSAGE library. Approximately 31%~34% of the total tags of each SAGE library were unique, while unique tags accounted for 45% of total tags of the HCC LongSAGE library (Table 2). When using SAGEmap to annotate these tags, we found that about 50% of unique tags in a SAGE library matched to specific genes (single match), 25~28% to multiple genes (multiple match) and 22~25% were novel tags that do not match to any known genes. The pattern was quite different in the LongSAGE library, with 43% of unique tags being single matched tags, 3.5% multiple matched tags and 53% novel tags. The result indicated that by extending the tag length from 14 to 21 bp, the specificity of SAGE tags was substantially improved. More than 70% of total tags in each library were single copy tags, while tags with high copy numbers (> 5 copies) account for less than 10% of the total tags (Table 3). This result is consistent with the fact that the majority of human genes are expressed at low levels [27].

**Table 2:** Tag counts of SAGE and LongSAGE libraries

	**Total**	**Unique** **(%)**	**Single matched** **(%)**	**Multiple matched** **(%)**	**Novel** **(%)**
**Normal Liver**	56,984	17,719 (31.1)	8,823 (49.8)	4,452 (25.1)	4,444 (25.1)
**HepG2**	47,149	15,827 (33.6)	7,956 (50.2)	4,427 (28.0)	3,444 (21.8)
**HCC**	63,800	20,313 (31.8)	10,048 (49.4)	5,375 (26.5)	4,890 (24.1)
**HCC LongSAGE**	51,632	23,093 (44.7)	9,973 (43.2)	801 (3.5)	12,319 (53.3)

**Table 3:** Frequency distributions of unique tags from the SAGE and LongSAGE libraries

**Tag copy No.**	**Normal Liver** **(%)**	**HepG2** **(%)**	**HCC** **(%)**	**HCC LongSAGE** **(%)**
**1**	12,842 (72.5)	11,223 (71.0)	14,477 (71.3)	18,171 (78.7)
**2**	2,123	2,015	2,495	2,205
**3**	872	847	1,002	947
**4**	447	429	558	469
**5–10**	864	837	1,083	823
**11–20**	291	248	364	256
**21–99**	231	166	268	180
**100 and >**	49	62	66	42

**Total**	17,719	15,827	20,313	23,093

### Correlation analysis of SAGE data

**Figure 1:**
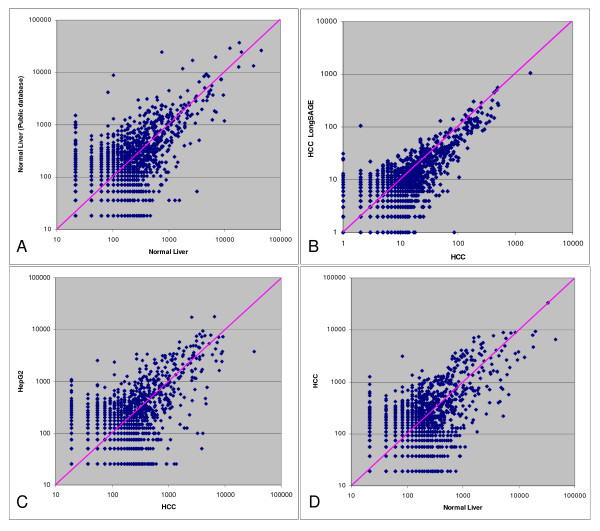
**Correlation analysis of SAGE and LongSAGE libraries**. (A) Correlation between normal liver SAGE data from public database  and our own normal liver data. Pearson's correlation coefficient 0.74. (B) Correlation between SAGE and LongSAGE data of HCC. Pearson's correlation coefficient 0.97. (C) Correlation between HCC and HepG2 SAGE data. Pearson's correlation coefficient 0.55. (D) Correlation between HCC and our own normal liver SAGE data. Pearson's correlation coefficient 0.70.

As one set of SAGE data of normal liver is available in the public Gene Expression Omnibus database (, accession number GSM785), we compared our data of normal liver with it to evaluate the correlation between these two independent libraries. The result indicated that the data from public database correlated well with our data, with Pearson's correlation coefficient 0.74 (Figure 1A).

We also compared the SAGE and LongSAGE data which were obtained from the same HCC tissue sample of the same patient. We extracted the 14-bp SAGE tags from the 21-bp LongSAGE tags. The converted SAGE library could be considered as a technical replicate. Totally 6,815 overlapping tags were detected between these two libraries. Thus among the 20,313 unique tags in the original SAGE library, only 33.5% of them are detected by the second library derived from LongSAGE tags. Most of the missed tags are low abundance tags for which detection by random sampling is a stochastic process. Another reason might be possible sequencing errors that resulted in many singleton tags. However, analysis of these overlapping tags showed remarkable consistency between these two libraries with Pearson's correlation coefficient 0.97 (Figure 1B). This result reassured us the reproducibility of SAGE method on measuring gene expression.

We further compared the data of HCC with that of HepG2 to find out how much the gene expression profile has changed in cell line comparing with the HCC tissue. The difference between HCC and HepG2 was very notable, with Pearson's correlation coefficient 0.55, even greater than difference between HCC and normal liver (Pearson's correlation coefficient 0.70) (Figure 1C and 1D). This result suggested that the gene expression profile of cell line has changed too much to be regarded as the reference of its corresponding tissue.

### Identification and functional analysis of genes differentially expressed between normal liver and HCC

**Figure 2:**
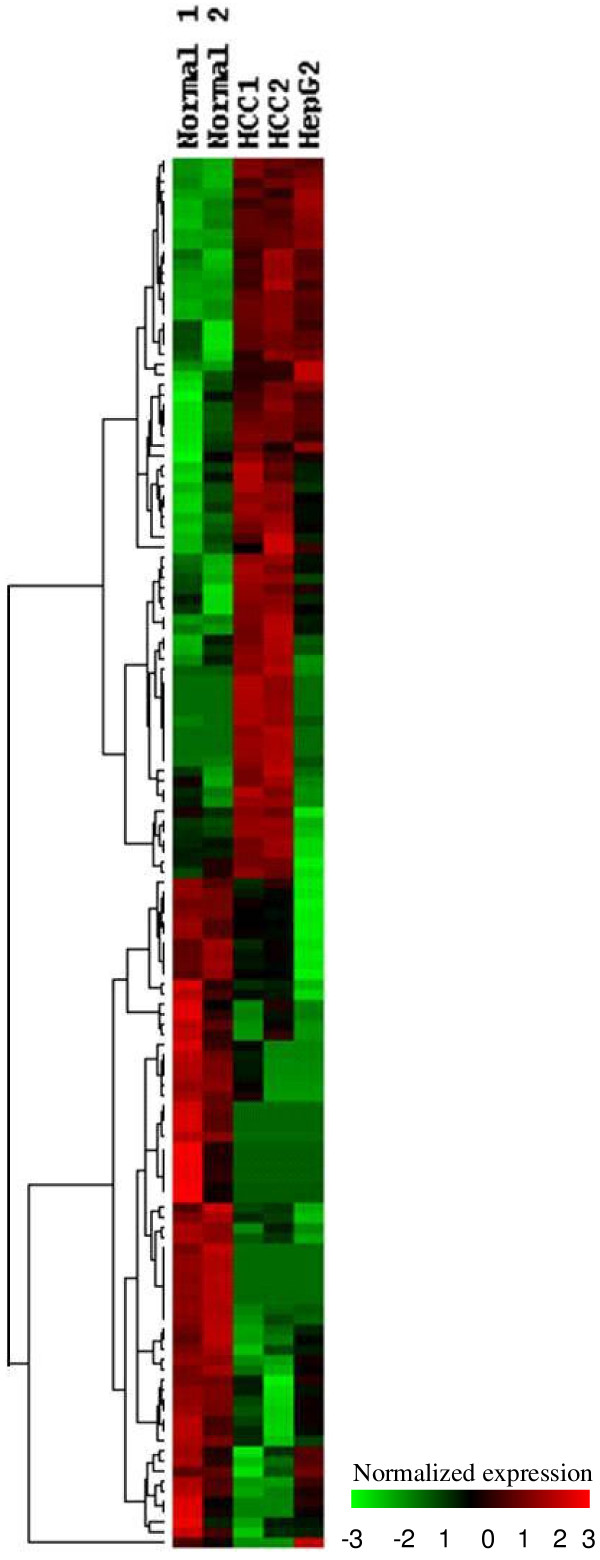
**Hierarchical clustering for genes differentially expressed in HCC**. For visual comparison, genes differentially expressed in HCC and normal liver were clustered by Treeview program. The expression pattern of these genes in HepG2 was also shown. The red color represents genes up-regulated, and the green color represents genes down-regulated. 1: normal liver, 2: normal liver from public database, 3: HCC SAGE, 4: HCC LongSAGE, 5: HepG2.

In order to identify genes differentially expressed between normal liver and HCC, we used the IDEG6 software to compare the normal liver library with both the HCC library and the second HCC library derived from LongSAGE. Only genes consistently up-regulated or down-regulated in both comparisons were considered to be differentially expressed. Totally 224 genes were identified to be differentially expressed, with the significance *p *< 0.05 and fold change > 3 (Additional file 1). Figure 2 shows the expression patterns of these altered genes in normal liver, HCC and HepG2. The top 20 up-regulated and down-regulated genes ranked by fold change were listed in Table 4 and Table 5 respectively. Consistent with previous results [18,28], our data also indicated that *GPC3 *gene was significantly up-regulated in HCC with fold change of 69. Among the 224 differentially expressed genes, the expression level of 104 genes was significantly higher in HCC than in normal liver (*p *< 0.05), while 120 genes was significantly lower in HCC (*p *< 0.05). In addition to these 224 annotated tags, our list of differentially expressed tags also includes 99 tags that could be matched to multiple genes, and 109 tags that were differentially expressed but could not be mapped to any known gene (data not shown). Some of these novel tags are extremely highly expressed in HCC. For example, the tag TAAGTTTGGG is detected 24 and 17 times in two HCC libraries but is not detected at all in the other two libraries. It will be of interest to further study these tags. In the current study, however, we will focus on the annotated tags.

**Table 4:** Top 20 genes up-regulated in HCC

**Tag**	**UniGene**	**Symbol**	**Name**	**Function**	**Fold Change**
ACCCGCCGGG	Hs.335106	ZFYVE1	Zinc finger, FYVE domain containing 1	Transport	81
GATTTCTTTG	Hs.435036	GPC3	Glypican 3	extracellular matrix	69
GCCAAGAATC	Hs.307720	DTNB	Dystrobrevin, beta	Calcium and Zinc ion binding	56
CTAACTAGTT	Hs.473583	NSEP1	Nuclease sensitive element binding protein 1	Response to external stimulus	50
GCTTGAATAA	Hs.116724	AKR1B10	Aldo-keto reductase family 1, member B10 (aldose reductase)	Steroid metabolism	47
GTGACCACGG	Hs.436980	GRIN2C	Glutamate receptor, ionotropic, N-methyl D-aspartate 2C	Ion transport	39
CTCATCCTAC	Hs.416049	TNPO2	Transportin 2 (importin 3, karyopherin beta 2b)	Protein transport	36
GATGTATGTG	Hs.461085		EST, strongly similar to NP_775842.1	Unclassified	36
ACCAGCACTC	Hs.436911	AMBP	Alpha-1-microglobulin/bikunin precursor	Signal Transduction	32
CCGACGGGCG	Hs.198161	PLA2G4B	Phospholipase A2, group IVB (cytosolic)	Signal Transduction	32
GGTCAGTCGG	Hs.409352	FLJ20701	Hypothetical protein FLJ20701	Unclassified	29
ATGTAAAAAA	Hs.429608	C5orf18	Chromosome 5 open reading frame 18	Unclassified	28
GACCCACCAT	Hs.436911	AMBP	Alpha-1-microglobulin/bikunin precursor	Signal Transduction	28
CAAGTTTGCT	Hs.439552	EEF1A1	Eukaryotic translation elongation factor 1 alpha 1	Signal Transduction	26
TTGTAAACTT	Hs.509226	FKBP3	FK506 binding protein 3, 25kDa	Cellular metabolism	25
GAAGGTGATC	Hs.112208	XAGE-1	X antigen family, member 1	Cellular metabolism	25
GGGACGAGTG	Hs.351316	TM4SF1	Transmembrane 4 L six family member 1	Unclassified	21
GAGGGTGGCG	Hs.473847	SH3BGR	SH3 domain binding glutamic acid-rich protein	Cellular metabolism	18
TAAATACAGT	Hs.221497	PRO0149	PRO0149 protein	Unclassified	18
GTGGATGGAC	Hs.6418	GPR175	G protein-coupled receptor 175	Organogenesis	17

**Table 5:** Top 20 genes down-regulated in HCC

**Tag**	**UniGene**	**Symbol**	**Name**	**Function**	**Fold Change**
GGCAGAGCCT	Hs.1955	SAA2	Serum amyloid A2	Response to external stimulus	56
TTAACTTTAT	Hs.368626	RTN1	Reticulon 1	Signal Transduction	56
TAAGCCCCGC	Hs.2899	HPD	4-hydroxyphenylpyruvate dioxygenase	Cellular metabolism	49
GACACCGAGG	Hs.333383	FCN3	Ficolin (collagen/fibrinogen domain containing) 3 (Hakata antigen)	Transport	44
TTGGCTAGAC	Hs.100914	Cep192	Centrosomal protein 192 kDa	Unclassified	40
TTCCAGAGGC	Hs.8821	HAMP	Hepcidin antimicrobial peptide	Cellular metabolism	38
GATCCCAACT	Hs.418241	MT2A	Metallothionein 2A	Metal Ion Binding	37
AAGGACGCCG	Hs.117367	SLC22A1	Solute carrier family 22 (organic cation transporter), member 1	Transport	37
AACTCACAGA	Hs.168718	AFM	Afamin	Transport	36
AGAATAAGAA	Hs.418167	ALB	Albumin	Regulation of body fluid (Blood coagulation)	26
GCGGCCCCCC	Hs.839	IGFALS	Insulin-like growth factor binding protein, acid labile subunit	Signal Transduction	24
TACAGCCTGT	Hs.406184	FGFR1OP2	FGFR1 oncogene partner 2	Unclassified	24
CTTGGGTTTT	Hs.355888	PLCB2	Phospholipase C, beta 2	Lipid metabolism	24
CACTTCAAGG	Hs.521903	LY6E	Lymphocyte antigen 6 complex, locus E	Signal Transduction	23
CCACCCCGAA	Hs.35052	TEGT	Testis enhanced gene transcript (BAX inhibitor 1)	Cell Death	22
CCATTACCTC	Hs.134958	RODH-4	Retinol dehydrogenase 16 (all-trans and 13-cis)	Lipid metabolism	21
AGTCTGGCCT	Hs.416707	ABCA4	ATP-binding cassette, sub-family A (ABC1), member 4	Response to external stimulus	21
CCAGCAAGAG	Hs.11900	ZGPAT	Zinc finger, CCCH-type with G patch domain	Unclassified	20
AGAACCTTCC	Hs.181244	HLA-A	Major histocompatibility complex, class I, A	Antigene presentation	19
CCCTTCTTTG	Hs.356368	HAO2	Hydroxyacid oxidase 2 (long chain)	Cellular metabolism	19

Functional analysis of the 224 differently expressed genes was performed using DAVID program. The 104 up-regulated genes were grouped into eight functional clusters and one unclassified cluster (Additional file 2). These genes activated in HCC were involved in biological processes of biosynthesis, cell proliferation, signal transduction, transport, response to external stimulus, and cellular metabolism *et al*. Genes related to biosynthesis include 10 ribosomal proteins. Consistent with our observation, increased expression pattern of ribosomal proteins in HCC were demonstrated by previous studies [29,30]. Genes participate in cell proliferation and signal transduction processes, such as *MCM7 *and *IGFBP1 *were also reported to be up-regulated in HCC by other groups [31,32].

The 120 down-regulated genes were assembled into seven functional Gene Ontology (level 3) categories including blood coagulation, cell death, cellular metabolism, transport, signal transduction *et al*., and one unclassified group (Additional file 3). We also tested the over-representation of GO terms of all levels in this list. The most significant cluster is 8 genes belongs to a subcategory related to acute inflammatory response with *p *< 0.0005 after Benjamini correction of multiple testing. This cluster includes *complement factor I *(*CFI*) and several genes (*C1S*, *C1QA*, and *C8B*) encoding subcomponents of complement component 1 and 8. Another significantly enhanced cluster includes 6 genes related to blood coagulation (*p *< 0.03). These results indicated the loss of liver function in HCC.

Taken together, functional classification of genes differentially expressed in HCC revealed dysregulated pathways which may contribute to the disease. Similar to our results, microarray studies carried out by other groups also indicated genes involved in protein biosynthesis and cell signaling were up-regulated in HCC, while genes expressed at lower level in HCC included complement proteins and clotting factors [33,34]. Thus, data obtained from SAGE and microarray methods could cross-validate each other and reveal some consistent events in HCC despite its heterogeneity.

### Validation of differentially expressed genes

To validate the differently expressed genes identified by SAGE, real-time quantitative RT-PCR analysis was performed in 20 pairs of HCC and adjacent nontumorous liver tissues. Totally 10 genes from SAGE data were selected for examination. *CCL20 *and *S100P *were significantly up-regulated in HepG2 but not in HCC data, while *ZFYVE1, PEG10, SGCE, XAGE-1, COL4A1, ZNF83, TNPO2 *and *TM4SF1 *were up-regulated in HCC data. SAGE tag abundance of these genes was listed in Table 6. *SGCE *was not included in the list of 224 differently expressed genes which were derived only from single matched tags, because the tag matched to *SGCE *was a multiple matched tag. Notably, *SGCE *locates on chromosome 7q21 together with *PEG10 *in a head-to-head manner, separated by only 115 base pairs between the 5' ends of these two genes. Among these genes, *CCL20 *and *PEG10 *have been demonstrated to be up-regulated in HCC by other groups [35-37], and were used as positive controls in our study. *XAGE-1, COL4A1, S100P *and *TM4SF1 *were observed to show elevated expression in various cancers, but the expression level of these genes in HCC has not been reported previously [38-43]. The possible association existing between *ZNF83, TNPO2, ZFYVE1 *and cancer was discussed in the present study for the first time.

**Table 6:** SAGE tag abundance of selected genes

		Tag Counts (Tag Per Million)			
Tag	Unigene	Normal liver	HCC	HCC-Long	HepG2
GAGGGTTTAG	CCL20	25	23	5	844
TACCTCTGAT	S100P	25	23	5	1091
GAAGGTGATC	XAGE-1	5	224	286	5
GACCGCAGGA	COL4A1	5	188	146	5
ACCCGCCGGG	ZFYVE1	5	553	1107	203
ATTTTGTCGT	ZNF83	5	188	122	5
CTCATCCTAC	TNPO2	5	315	427	5
GAAGTTATAA	TM4SF1	25	169	685	499
GAAGTTATAA	PEG10	5	133	216	128
TTGGCAGTAT	SGCE; DDX43	25	242	263	30

**Figure 3:**
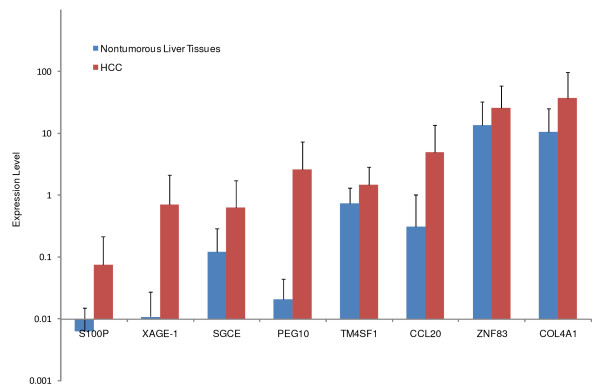
**Real-time quantitative RT-PCR validation of SAGE data**. Expression level of candidate genes identified to be up-regulated in HCC or HepG2 by SAGE data was examined in 20 pairs of HCC and corresponding nontumorous liver tissues by quantitative RT-PCR. Increased expression level in HCC was observed in CCL20, COL4A1, XAGE-1, PEG10, SGCE, S100P, TM4SF1 and ZNF83 genes (*p *< 0.05). Red bars represent expression level of HCC tissues, blue bars represent nontumorous liver tissues, and horizontal bars represent SD values.

**Figure 4:**
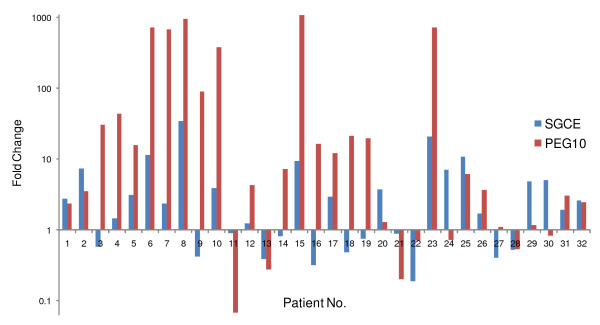
**Similar expression patterns of PEG10 and SGCE in HCC patients**. Expression level of PEG10 and SGCE was detected by real-time quantitative RT-PCR in 32 pairs of HCC and corresponding nontumorous liver tissues. The fold changes between HCC and nontumorous liver were represented by the height of bars (logarithmic scale). Red bars represent PEG10, and blue bars represent SGCE. These two genes showed similar expression patterns in 23 out of 32 patients, with Pearson's correlation coefficient 0.71.

By real-time quantitative RT-PCR analysis, increased expression was confirmed in 8 of these 10 genes. As shown in Figure 3, *CCL20, S100P, PEG10, SGCE, XAGE-1, COL4A1, ZNF83*, and *TM4SF1 *was significantly up-regulated in HCC compared to the corresponding nontumorous liver (*p *< 0.05). Expression level of *PEG10 *and *SGCE *was further examined in more samples (totally 32 pairs of HCC and corresponding nontumorous liver). Similar expression patterns of these two genes were observed in HCC, with Pearson's correlation coefficient 0.71 (Figure 4). No significant differences in expression level of *TNPO2 *and *ZFYVE1 *was detected between HCC and adjacent nontumorous liver, reflecting the heterogeneous feature of HCC. These results further validated the quantitative data of SAGE profiles and provided novel candidate genes which may help to illustrate the molecular mechanism of hepatocarcinogenesis.

## Discussion

In this study, we applied SAGE to analyze gene expression profiles of normal liver, HepG2 and HCC on the genome scale. LongSAGE was also carried out to analyze the same HCC sample. From our results we found that the proportion of unique tags was higher in the HCC LongSAGE library (45%) than in the SAGE library (32%), suggesting that LongSAGE is definitely more efficient in detecting unique tags and tag-to-gene mapping than SAGE. Among the 20,313 unique tags in the HCC SAGE library, 6,815 (33.5%) of them were detected by LongSAGE tags with high correlation (Pearson's correlation 0.97). This indicated that the data of SAGE is repeatable even by LongSAGE. Thus it is reasonable that Pearson's correlation observed between our normal liver data and those in the public database was as high as 0.74 although they were obtained from totally independent experiments. We could draw a conclusion from these results that comparison among SAGE data produced by different labs is practical and reliable because of the high repeatability of SAGE technique.

It is not to our surprise that HepG2, a cultured hepatocellular carcinoma cell line, exhibited a different gene expression pattern compared to the pattern of HCC. Within passage cells, a lot of changes might have occurred on the genome scale which would subsequently affect the gene expression level. Relatively low correlation (Pearson's correlation coefficient 0.55) was observed between HepG2 and HCC in our SAGE data set. This result suggested that the expression profile of cultured cells should be cautiously used as reference of the corresponding tissue. However, analysis of HepG2 data still could provide some useful clues in identifying genes differentially expressed between normal liver and HCC. For example, increased expression of both *CCL20 *and *S100P *genes were detected in HCC by quantitative RT-PCR, although they were found to be significantly up-regulated only in HepG2 but not in HCC SAGE data. Consistent with our result, recent studies carried out by Rubie *et al *indicated that *CCL20 *was activated in HCC and supposed to be involved in hepatocarcinogenesis [35,36]. Up-regulation of *S100P *was reported in breast cancer, prostate cancer and early-stage non-small cell lung cancer [38-40], and it was also suggested to be a key factor in the aggressiveness of pancreatic cancer and promote cancer growth, survival and invasion [41]. Our results indicated that *S100P *was up-regulated in HCC, suggesting a possible role of *S100P *may play in liver cancer.

Enhanced expression of *COL4A1, TM4SF1, XAGE-1, ZNF83, PEG10 *and *SGCE *in HCC was detected by our SAGE data, and further validated by real-time quantitative RT-PCR. Potential roles of *COL4A1, TM4SF1*, and *XAGE-1 *in various cancers have been demonstrated including oral squamous cell carcinomas, gastric cancer, breast cancer and lung cancer *et al*, but not liver cancer [42-44]. Our results gave a clue to the probability that these genes may also play a role in HCC pathogenesis. The maternally imprinted gene *PEG10 *was recently identified as a potential biomarker for HCC diagnosis, and genomic gain accounts for the major cause of its up-regulation in HCC [37]. *SGCE *was also a maternally imprinted gene, locating on chromosome 7q21 as close as 115 base pairs apart from *PEG10 *in a head-to-head manner. Due to the original SAGE tag TTGGCAGTAT matched to *SGCE *and *DDX43 *(*DEAD box polypeptide 43*) gene simultaneously, it was not included in our later analysis of differentially expressed genes in HCC which were derived only from single matched SAGE tags. However, *PEG10 *and *SGCE *were demonstrated to be up-regulated in a parallel way in B-cell chronic lymphocytic leukemia patients [45]. We examined the expression of these two genes in HCC and found that *PEG10 *and *SGCE *show similar expression patterns with close correlation (Pearson's correlation coefficient 0.71), pointing towards the possibility that these two genes may share common mechanism of deregulation in HCC. Whether *PEG10 *and *SGCE *are co-regulated via chromosome amplification at the 7q21 locus or their common promoter region is under investigating by our further study.

## Conclusion

In summary, this study depicted the expression profile of HCC using both SAGE and LongSAGE techniques, which would provide valuable clues for understanding of molecular mechanism of HCC. Further investigations are needed for the candidate genes suggested by this study, especially for *PEG10 *and *SGCE *genes locating together on chromosome 7q21, and for many of the tags highly expressed in HCC but could not be matched to any known genes.

## Abbreviations

HCC: hepatocellular carcinoma; SAGE: serial analysis of gene expression

## Competing interests

The authors declare that they have no competing interests.

## Authors' contributions

HD designed the experiments, participated in most experiments and drafted the manuscript. XJG performed the bioinformatics analyses. YS, LLC and YLK generated expression profiles of normal liver, HCC and HepG2. XBM and LT performed the qPCR experiments. HYZ and HY participated in collecting clinical tissue samples. HYW participated in analyzing the data. GPZ and WRJ were responsible for data analysis, manuscript drafting and revision.

## Pre-publication history

The pre-publication history for this paper can be accessed here:



## Supplementary Material

Additional file 1 **List of genes differentially expressed in HCC vs normal liver.** 224 genes were identified to be differentially expressed in HCC vs normal liver, with the significance p < 0.05 and fold change > 3. Click here for file

Additional file 2 **Functional classification of genes up-regulated in HCC. **104 genes up-regulated in HCC were grouped into eight functional clusters and one unclassified cluster. Click here for file

Additional file 3 **Functional classification of genes down-regulated in HCC.** 120 genes down-regulated in HCC were assembled into seven functional clusters and one unclassified cluster. Click here for file
